# Low-PAPR ASE-DMT Using Constellation Extension for Optical Wireless Communications

**DOI:** 10.3390/s25165109

**Published:** 2025-08-17

**Authors:** Yue Wu, Yiding Li, Baolong Li

**Affiliations:** 1 Changwang School of Honors, Nanjing University of Information Science and Technology, Nanjing 210044, China; wytg@nuist.edu.cn; 2 School of Electronic and Information Engineering, Nanjing University of Information Science and Technology, Nanjing 210044, China; lydcg@nuist.edu.cn

**Keywords:** optical wireless communication (OWC), augmented spectral efficiency discrete multitone (ASE-DMT), peak-to-average power ratio, constellation extension

## Abstract

In the realm of optical wireless communication (OWC), augmented spectral efficiency discrete multitone (ASE-DMT) has been widely recognized as a promising modulation due to its outstanding spectral efficiency and high power efficiency. However, ASE-DMT exhibits an inherently high peak-to-average power ratio (PAPR), which exacerbates error propagation and leads to a substantial transmission performance degradation in the successive interference cancellation (SIC) receiver of ASE-DMT. Therefore, a novel low-PAPR ASE-DMT scheme (LP-ASE-DMT) is proposed in the paper. Given the intricate multi-depth signal superposition of ASE-DMT, a progressive multi-level constellation extension algorithm is developed to effectively suppress the PAPR of the transmitted signal, while simultaneously achieving much lower computational complexity compared to conventional constellation extension schemes. Furthermore, a dedicated receiver architecture is designed for LP-ASE-DMT, in which a low-complexity modulo operation is employed to eliminate the impact of constellation extension without incurring significant additional receiver complexity. The effectiveness of the proposed LP-ASE-DMT scheme is validated through simulation, revealing a substantial mitigation of PAPR compared to its counterparts. This improvement notably strengthens the system’s robustness to nonlinear impairments. Consequently, LP-ASE-DMT enjoys superior performance across multiple metrics, including bit error rate (BER), power efficiency, and spectral efficiency.

## 1. Introduction

With the rapid advancement of information technology, traditional radio-frequency (RF) wireless communication is confronted with the escalating challenge of spectrum scarcity. Against this background, optical wireless communication (OWC) has emerged as a promising technology and has attracted considerable attention. Utilizing optical carriers for data transmission, OWC enjoys numerous advantages, including inherent immunity to electromagnetic interference, ultra-high bandwidth, enhanced security, and so on [1,2]. Admittedly, OWC also suffers from some limitations, such as strong reliance on line-of-sight (LoS) transmission, sensitivity to weather conditions, and so on. Furthermore, when employing light-emitting diode as the optical source, the limited coherence of the emitted light imposes significant constraints on the achievable transmission distance. Nevertheless, given the spectrum scarcity of RF, OWC is widely recognized as an effective complementary technology, which demonstrates significant potential in a wide range of application domains, including mobile communications, internet of things (IoT), and underwater communications [3,4,5]. In particular, OWC demonstrates exceptional applicability in scenarios characterized by stringent electromagnetic compatibility requirements, such as hospitals, nuclear power plants, and aerospace environments, where conventional RF systems may pose safety risks or experience significant performance limitations [6].

Within optical wireless communication (OWC) systems, orthogonal frequency division multiplexing (OFDM) has emerged as a prominent multicarrier scheme [7,8]. As OWC typically relies on intensity modulation and direct detection (IM/DD), the transmitted signal must be strictly real and non-negative [9,10]. Consequently, various tailored optical OFDM (O-OFDM) techniques have been developed to accommodate these constraints. Classical schemes include direct current-biased optical OFDM (DCO-OFDM), asymmetrically clipped optical OFDM (ACO-OFDM), and pulse-amplitude modulation discrete multitone (PAM-DMT), and so on [10,11]. Although these exciting works demonstrate low implementation complexity, they suffer from reduced power efficiency or suboptimal spectral efficiency. To overcome these limitations, several enhanced variants of O-OFDM have been devised, such as hybrid ACO-OFDM (HACO-OFDM), layered ACO-OFDM (LACO-OFDM), and augmented spectral efficiency discrete multitone (ASE-DMT) [11,12]. In these advanced schemes, multiple O-OFDM signals are combined in a superimposed manner to improve spectral efficiency, while simultaneously preserving high power efficiency. Notably, ASE-DMT is capable of achieving spectral efficiency comparable to that of DCO-OFDM as the number of modulation depths increases, while offering significantly higher power efficiency [13]. Benefiting from its superior trade-off between spectral and power efficiency, ASE-DMT has attracted substantial interest in the field of OWC [14,15].

OFDM inherently suffers from a high PAPR, which renders it highly susceptible to the severe nonlinear distortions in non-ideal transmission scenarios [16]. Under such circumstances, the OFDM system could suffer from order-of-magnitude degradation in bit-error rate (BER) performance. Consequently, the pursuit of effective PAPR reduction has become a pivotal research direction in OFDM [17]. Moreover, the high PAPR imposes even more stringent challenges on ASE-DMT architectures employing multiple-depth signal superposition, further exacerbating the system’s vulnerability to nonlinear distortions. This is primarily attributed to the fact that ASE-DMT receivers typically rely on a successive interference cancellation (SIC) strategy to detect signals depth by depth. Under such a framework, nonlinear distortions can trigger severe error propagation across layers, thereby leading to significant degradation in transmission performance [14]. As a result, PAPR reduction is of paramount importance for the practical implementation of ASE-DMT.

To address the problem of high PAPR, various PAPR reduction schemes have been proposed, including clipping, selective mapping (SLM), partial transmit sequences (PTS), tone reservation (TR), constellation extension, and so on [18,19,20,21]. Among these schemes, direct clipping enjoys its simplicity of implementation, but introduces severe signal distortion and deteriorates overall system performance [22]. SLM and PTS reduces the PAPR by applying phase rotation to the entire block and sub-blocks, respectively. However, both schemes incur high computational complexity due to multiple IFFT operations and necessitate the transmission of side information, thereby introducing additional communication overhead. TR achieves PAPR mitigation by reserving a dedicated subset of subcarriers to generate the peak-cancelling signal. Consequently, PAPR reduction via SLM, PTS, and TR inherently incurs a penalty in spectral efficiency [23]. In contrast, constellation extension offers a compelling alternative that avoids the drawbacks of the aforementioned schemes while achieving significant PAPR reduction. To date, numerous works have been conducted on the constellation-extension-based PAPR reduction in OWC [24,25,26,27]. Most of these works are designed for the conventional O-OFDM schemes characterized by relatively simple time-domain architecture, and thus cannot be directly applied to ASE-DMT with multi-depth signal superposition. There are a few works investigating the design of the constellation-extension-based PAPR reduction for hybrid optical OFDM (O-OFDM) schemes [26,27]. However, these works primarily focus on the subcarrier selection through sophisticated convex optimization techniques. Meanwhile, they are only applicable to single-level constellation extension, thereby failing to fully exploit the available degrees of freedom. As a result, there remains substantial room for improvement in terms of PAPR reduction performance. Therefore, the development of an efficient constellation extension-based PAPR reduction strategy tailored for ASE-DMT remains an urgent and unresolved research challenge.

Against this background, a novel low-PAPR ASE-DMT (LP-ASE-DMT) scheme based on constellation extension is proposed. The main contributions of the paper are summarized as follows

- Given the intricate multi-layered superposition structure inherent in ASE-DMT, a progressive constellation extension algorithm is developed to effectively reduce the PAPR in the proposed LP-ASE-DMT. Compared to the conventional schemes, the proposed LP-ASE-DMT enables multi-level constellation extension, thereby enhancing the degrees of freedom to achieve more effective PAPR suppression. Moreover, the proposed scheme relies on a low-complexity search mechanism, which does not requires the computationally intensive convex optimization.
- Furthermore, a corresponding receiver architecture for LP-ASE-DMT is designed, wherein a low-complexity modulo operation is employed to eliminate the impact of constellation extension. Notably, this design does not significantly increase the overall receiver complexity compared to the original ASE-DMT architecture.
- Simulation results demonstrate that the proposed LP-ASE-DMT achieves a substantial PAPR reduction of up to 5.5 dB at a probability of $1\times 10^{-4}$, significantly outperforming the conventional ASE-DMT scheme. Benefiting from its reduced PAPR, the proposed method exhibits superior performance under nonlinear transmission conditions, including lower BER, improved power efficiency, and enhanced spectral efficiency, thereby considerably improving the reliability and practicality of ASE-DMT in nonlinear OWC systems.

The remainder of this paper is organized as follows: Section 2 presents the theoretical foundation of ASE-DMT. Section 3 describes the design of the proposed LP-ASE-DMT. Section 4 presents the receiver of the proposed LP-ASE-DMT. Section 5 provides simulation results, and Section 6 concludes the paper.

## 2. Augmented Spectral Efficiency Discrete Multitone

In ASE-DMT, PAM-DMT signals from multiple depths are combined for concurrent transmission to enhance spectral efficiency. Let $X_{k}^{\left(d\right)}$ represent the frequency-domain signal corresponding to the *d*-th depth. At the first depth, data symbols are embedded into the imaginary parts of the subcarriers. Consequently, the analytical expression of the frequency-domain signal for the first depth is formulated as follows:

(1) $$X_{k}^{\left(1\right)}=\left\{\begin{array}{cc} jP_{k}^{\left(1\right)}, & k=1,2,\ldots ,\frac{N}{2}-1,\\ -jP_{N-k}^{\left(1\right)}, & k=\frac{N}{2}+1,\frac{N}{2}+2,\ldots ,N-1,\\ 0, & \mathrm{otherwise}, \end{array}\right.$$

where $P_{k}^{\left(1\right)}$ is the PAM symbol modulated on the *k*-th subcarrier of the first depth. Furthermore, the signals in subsequent depths exploit the real part of the subcarriers for data transmission. For the *d*-th depth with d≥2, the frequency-domain signal is written as

(2) $$X_{k}^{\left(d\right)}=\left\{\begin{array}{cc} P_{k}^{\left(d\right)}, & k=2^{d-1}\left(2i+1\right)\;\mathrm{and}\;k<\frac{N}{2},\\ P_{N-k}^{\left(d\right)}, & k=2^{d-1}\left(2i+1\right)\;\mathrm{and}\;k>\frac{N}{2},\\ 0, & \mathrm{otherwise} \end{array}\right.$$

where $P_{k}^{\left(d\right)}$ denotes the PAM symbol modulated on the *k*-th subcarrier at the *d*-th depth. By inputting the signal $X_{k}^{\left(d\right)}$ into the IFFT module, the corresponding time-domain signal can be generated, denoted as $x_{n}^{\left(d\right)}$. The signal $x_{n}^{\left(d\right)}$ can be further processed by using direct clipping operation to generate a non-negative signal, thereby ensuring power-efficient transmission. Meanwhile, ASE-DMT adopts a hierarchical superposition transmission strategy, where signals from multiple depths are jointly transmitted to enhance spectral efficiency. Specifically, the ASE-DMT signal can be formulated as

(3) $$y_{n}=\sum_{d=1}^{D}{\left\lfloor x_{n}^{\left(d\right)}\right\rfloor }_{c},$$

where ${\left\lfloor \cdot \right\rfloor }_{c}$ represents the direct clipping operator, and *D* is the total number of superimposed time-domain signals. Owing to the fact that the clipping noise generated at a specific depth propagates to the subsequent layers without corrupting the symbol transmission at the current depth, ASE-DMT adopts a SIC framework for depth-wise signal detection. In this framework, the PAM symbols corresponding to the current depth are first detected and subsequently leveraged to estimate and reconstruct the associated clipping noise. The reconstructed noise is then subtracted from the corresponding subcarriers of the deeper PAM-DMT to effectively suppress the interference affecting the subsequent depths. This iterative process is carried out across all depths until the complete set of PAM symbols is accurately recovered.

## 3. Low-PAPR ASE-DMT

### 3.1. Design of Low-PAPR ASE-DMT

Due to the adoption of the SIC-based receiver architecture, the ASE-DMT system is susceptible to error propagation, which further exacerbates its intrinsic limitation of high PAPR. Specifically, nonlinear distortion-induced errors resulting from high PAPR in the preceding depths may propagate to subsequent depths, thereby significantly impairing signal detection performance. To address this issue, an effective PAPR reduction technique tailored for ASE-DMT is proposed based on constellation extension in this section.

**Figure 1:**
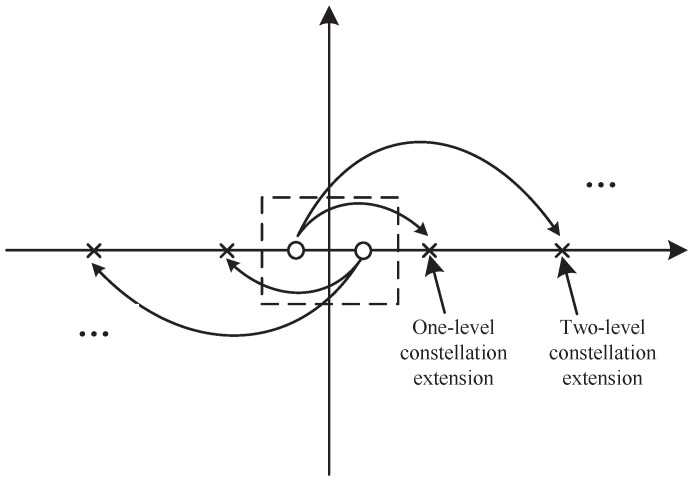
Schematic diagram of constellation extension of PAM.

To reduce the PAPR of ASE-DMT, the PAM symbols can be subjected to multi-level constellation extension. The constellation extension of PAM is illustrated in Figure 1. The frequency-domain signal of the first depth in ASE-DMT with constellation extension can be formulated as

(4) $${\hat{X}}_{k}^{\left(1\right)}=\left\{\begin{array}{cc} j\left[P_{k}^{\left(1\right)}-q_{k}^{\left(1\right)}\;\mathrm{sgn}\left(P_{k}^{\left(1\right)}\right)\Delta_{k}^{\left(1\right)}\right], & k=1,2,\ldots ,\frac{N}{2}-1,\\ -j\left[P_{N-k}^{\left(1\right)}-q_{N-k}^{\left(1\right)}\;\mathrm{sgn}\left(P_{N-k}^{\left(1\right)}\right)\Delta_{N-k}^{\left(1\right)}\right], & k=\frac{N}{2}+1,\frac{N}{2}+2,\ldots ,N-1,\\ 0, & \mathrm{otherwise}. \end{array}\right.$$

Here, ${\hat{X}}_{k}^{\left(1\right)}$ denotes the constellation-extended signal at the *k*-th subcarrier of the first depth. The variable $q_{k}^{\left(1\right)}\geq 0$ denotes an integer-valued decision parameter that determines the extent of constellation extension. Specifically, $q_{k}^{\left(1\right)}=0$ indicates that no constellation extension is applied to the *k*-th subcarrier, whereas $q_{k}^{\left(1\right)}>0$ corresponds to a multi-level extension with an extension depth of $q_{k}^{\left(1\right)}$ levels. The function $\mathrm{sgn}(P_{k}^{\left(1\right)})$ denotes the sign of the symbol $P_{k}^{\left(1\right)}$, and the parameter $\Delta_{k}^{\left(1\right)}=\rho d_{k}^{\left(1\right)}M_{k}^{\left(1\right)}$ defines the length of a single-level constellation extension, where ρ≥1 is an expansion factor. The term $d_{k}^{\left(1\right)}$ indicates the minimum distance between adjacent PAM constellation points on the *k*-th subcarrier of the first-depth signal, and $M_{k}^{\left(1\right)}$ denotes the modulation order of the PAM symbol.

For the *d*-th (d≥2) depth, the frequency-domain signal with multi-level constellation extension can be written as

(5) $${\hat{X}}_{k}^{\left(d\right)}=\left\{\begin{array}{cc} P_{k}^{\left(d\right)}-q_{k}^{\left(d\right)}\;\mathrm{sgn}\left(P_{k}^{\left(d\right)}\right)\Delta_{k}^{\left(d\right)}, & k=2^{d-2}\left(2i+1\right)\;\mathrm{and}\;k<\frac{N}{2},\\ P_{N-k}^{\left(d\right)}-q_{N-k}^{\left(d\right)}\;\mathrm{sgn}\left(P_{N-k}^{\left(d\right)}\right)\Delta_{N-k}^{\left(d\right)}, & k=2^{d-2}\left(2i+1\right)\;\mathrm{and}\;k>\frac{N}{2},\\ 0, & k\;\mathrm{otherwise}. \end{array}\right.$$

Here, $i=0,1,\ldots ,N/2^{d}-1$, ${\hat{X}}_{k}^{\left(d\right)}$ denotes the frequency-domain signal at the *k*-th subcarrier of the *d*-th depth. The variable $q_{k}^{\left(d\right)}\geq 0$ is an integer-valued decision parameter. Similarly, $q_{k}^{\left(d\right)}=0$ indicates no constellation extension, while $q_{k}^{\left(d\right)}=q>0$ represents that a *q*-level constellation extension is applied. The operator $\mathrm{sgn}\left(P_{k}^{\left(d\right)}\right)$ denotes the sign of the symbol $P_{k}^{\left(d\right)}$, and $\Delta_{k}^{\left(d\right)}=\rho d_{k}^{\left(d\right)}M_{k}^{\left(d\right)}$ denotes the length of a single-level extension. The term $d_{k}^{\left(d\right)}$ denotes the minimum distance between adjacent constellation points on the *k*-th subcarrier of the *d*-th depth, and $M_{k}^{\left(d\right)}$ represents the modulation order of the PAM constellation.

Since the PAPR performance is ultimately determined by the time-domain signal peaks, it is difficult to directly analyze the PAPR characteristics of the ASE-DMT signal from its frequency-domain structure. Therefore, in order to investigate the impact of constellation extension on the time-domain signal peaks, the frequency-domain signal is further transformed into its time-domain representation. Given the Hermitian symmetry, the time-domain signal $x_{n}^{\left(1\right)}$ corresponding to ${\hat{X}}_{k}^{\left(1\right)}$ can be expressed as

(6) $$\begin{array}{cc} x_{n}^{\left(1\right)}= & \frac{1}{N}\sum_{k=0}^{N-1}{\hat{X}}_{k}^{\left(1\right)}e^{j2\pi \frac{kn}{N}}=\frac{1}{N}\sum_{k=1}^{N/2-1}j\left[P_{k}^{\left(1\right)}-q_{k}^{\left(1\right)}\mathrm{sgn}\left(P_{k}^{\left(1\right)}\right)\Delta_{k}^{\left(1\right)}\right]e^{j2\pi \frac{kn}{N}}\\ \; & +\frac{1}{N}\sum_{k=1}^{N/2-1}\left[-j\left(P_{k}^{\left(1\right)}-q_{k}^{\left(1\right)}\mathrm{sgn}\left(P_{k}^{\left(1\right)}\right)\Delta_{k}^{\left(1\right)}\right)\right]e^{j2\pi \frac{(N-k)n}{N}}\\ = & x_{n}^{\left(1\right)}+\frac{1}{N}\sum_{k=1}^{N/2-1}2q_{k}^{\left(1\right)}\mathrm{sgn}\left(P_{k}^{\left(1\right)}\right)\Delta_{k}^{\left(1\right)}\cos\left(\frac{2\pi nk}{N}\right), \end{array}$$

It can be observed that the constellation-extended signal ${\hat{x}}_{n}^{\left(1\right)}$ consists of the original time-domain signal $x_{n}^{\left(1\right)}$ and the additive component induced by the constellation extension. To further characterize this, we define $\Gamma_{k,n}^{\left(1\right)}$ as the time-domain signal corresponding to a single-level constellation extension length $\Delta_{k}^{\left(1\right)}$, which is formulated as

(7) $$\Gamma_{k,n}^{\left(1\right)}=\frac{2}{N}\Delta_{k}^{\left(1\right)}\cos\left(\frac{2\pi nk}{N}\right),\;k=1,2,\ldots ,N/2-1.$$

Accordingly, the time-domain signal resulting from a single-level constellation extension on the *k*-th subcarrier of the first depth can be expressed as

(8) $$w_{k,n}^{\left(1\right)}=\mathrm{sgn}\left(P_{k}^{\left(1\right)}\right)\Gamma_{k,n}^{\left(1\right)},\;k=1,2,\ldots ,N/2-1.$$

Since $\Gamma_{k,n}^{\left(1\right)}$ remains invariant across different OFDM symbols, it can be precomputed and stored. Consequently, for different OFDM symbols, the time-domain signal corresponding to constellation extension, denoted as $w_{k,n}^{\left(1\right)}$, can be efficiently derived by multiplying the subcarrier’s polarity (i.e., the sign of the transmitted symbol) with $\Gamma_{k,n}^{\left(1\right)}$. This approach significantly reduces the computational complexity of time-domain reconstruction for constellation extension.

Analogously, for the *d*-th (d≥2) depth, the time-domain signal after constellation extension is given by

(9) $$\begin{array}{cc} {\hat{x}}_{n}^{\left(d\right)}= & \frac{1}{N}\sum_{k=0}^{N-1}{\hat{X}}_{k}^{\left(d\right)}e^{j2\pi \frac{kn}{N}}\\ = & \frac{1}{N}\sum_{i=0}^{N/2^{d}-2}j\left[P_{2^{d-2}\left(2i+1\right)}^{\left(d\right)}-q_{2^{d-2}\left(2i+1\right)}^{\left(d\right)}\mathrm{sgn}\left(P_{2^{d-2}\left(2i+1\right)}^{\left(d\right)}\right)\Delta_{2^{d-2}\left(2i+1\right)}^{\left(d\right)}\right]e^{j2\pi \frac{2^{d-2}\left(2i+1\right)n}{N}}\\ \; & +\frac{1}{N}\sum_{i=0}^{N/2^{d}-1}-j\left[P_{2^{d-2}\left(2i+1\right)}^{\left(d\right)}-q_{2^{d-2}\left(2i+1\right)}^{\left(d\right)}\mathrm{sgn}\left(P_{2^{d-2}\left(2i+1\right)}^{\left(d\right)}\right)\Delta_{2^{d-2}\left(2i+1\right)}^{\left(d\right)}\right]e^{j2\pi \frac{[N-2^{d-2}\left(2i+1\right)]n}{N}}\\ = & x_{n}^{\left(d\right)}+\frac{1}{N}\sum_{i=0}^{N/2^{d}-1}2q_{2^{d-2}\left(2i+1\right)}^{\left(d\right)}\mathrm{sgn}\left(P_{2^{d-2}\left(2i+1\right)}^{\left(d\right)}\right)\Delta_{2^{d-2}\left(2i+1\right)}^{\left(d\right)}\sin\left[\frac{2\pi n2^{d-2}\left(2i+1\right)}{N}\right]. \end{array}$$

To proceed, we define $\Gamma_{2^{d-2}\left(2i+1\right),n}^{\left(d\right)}$ as the time-domain signal corresponding to the constellation extension length $\Delta_{2^{d-2}\left(2i+1\right)}^{\left(d\right)}$ at the $2^{d-2}\left(2i+1\right)$-th subcarrier of the *d*-th (d≥2) depth. Furthermore, the signal $\Gamma_{2^{d-2}\left(2i+1\right),n}^{\left(d\right)}$ can be calculated as

(10) $$\Gamma_{i,n}^{\left(d\right)}=\frac{2}{N}\Delta_{2^{d-2}\left(2i+1\right)}^{\left(d\right)}\sin\left(\frac{2\pi n2^{d-2}\left(2i+1\right)}{N}\right).$$

Therefore, the time-domain signal of a single-level constellation extension at the $2^{d-2}\left(2i+1\right)$-th subcarrier is expressed as

(11) $$w_{i,n}^{\left(d\right)}=\mathrm{sgn}\left(p_{2^{d-2}\left(2i+1\right)}^{\left(d\right)}\right)\Gamma_{2^{d-2}\left(2i+1\right),n}^{\left(d\right)}.$$

In the process of constellation extension, it is essential to the integer-valued decision variables $q_{k}^{\left(1\right)}$ and $q_{2^{d-2}\left(2i+1\right)}^{\left(d\right)}$. However, optimizing these variables for PAPR suppression leads to a highly complex and non-convex integer programming problem, which is notoriously difficult to solve due to its high-dimensional, discrete, and nonlinear structure. To address the challenge, a stepwise constellation extension algorithm is proposed. In each iteration, the algorithm strategically identifies a subcarrier that necessitates extension and performs a single-level constellation extension on its associated time-domain signal. The extended signal subsequently serves as a reference foundation for the next round of subcarrier selection and extension. By adopting a recursive and cyclic optimization framework, the proposed method enables progressive realization of multi-level constellation extension, thereby substantially mitigating the PAPR of ASE-DMT.

Specifically, in the *m*-th iteration, the time-domain signal of the *d*-th depth after constellation extension is denoted as ${\hat{x}}_{n}^{\left(d,m\right)}$, which is recursively derived based on the extended signal ${\hat{x}}_{n}^{\left(d,m-1\right)}$ in the preceding iteration. The constellation extension for the *d*-th depth is first analyzed. Let $i_{d}$ denote the index of the subcarrier selected for extension at the *d*-th depth, where $i_{1}=1,2,\ldots ,N/2-1$ while $i_{d}=0,1,\ldots ,N/2^{d}-1$ for d≥2. The time-domain signal after performing a single-level constellation extension can be expressed as

(12) $${\tilde{x}}_{i_{d},n}^{\left(d,m\right)}={\hat{x}}_{n}^{\left(d,m-1\right)}+w_{i_{d},n}^{\left(d\right)}.$$

Furthermore, the constellation-extended ASE-DMT signal corresponding to ${\tilde{x}}_{i_{d},n}^{\left(d,m\right)}$ can be expressed as

(13) $${\tilde{y}}_{i_{d},n}^{\left(d,m\right)}=\sum_{l\neq d}{\left\lfloor {\hat{x}}_{n}^{\left(l,m-1\right)}\right\rfloor }_{c}+{\left\lfloor {\tilde{x}}_{i_{d},n}^{\left(d,m\right)}\right\rfloor }_{c},\;n=0,1,\ldots ,N-1.$$

To identify the optimal constellation extension at the *d*-th depth, the candidate subcarrier that yields the minimum peak amplitude is selected, which is given by

(14) $$b_{d,m}=\min_{i_{d}}\max_{n}\left\{{\tilde{y}}_{i_{d},n}^{\left(d,m\right)}\right\}.$$

The index of the subcarrier corresponding to the minimum peak amplitude is denoted by $i_{d}^{*}$. Subsequently, among all candidates $b_{1,m},b_{2,m},\ldots ,b_{D,m}$, the minimum one is selected as the solution of the constellation extension in the *m*-th iteration, i.e.,

(15) $$b_{m}=\min_{d}\left\{b_{d,m}\right\}.$$

The depth index corresponding to the minimum peak amplitude $b_{m}$ is denoted by $d^{*}$. Accordingly, the constellation extension is performed on the $i_{d^{*}}^{*}$-th subcarrier at depth $d^{*}$. Subsequently, the time-domain signal ${\hat{x}}_{n}^{\left(d,m\right)}$ is updated as follows:

(16) $${\hat{x}}_{n}^{\left(d,m\right)}=\left\{\begin{array}{cc} {\hat{x}}_{n}^{\left(d,m-1\right)}, & d\neq d^{*},\\ {\tilde{x}}_{i_{d^{*}},n}^{\left(d^{*},m\right)}, & d=d^{*}. \end{array}\right.$$

**Figure 2:**
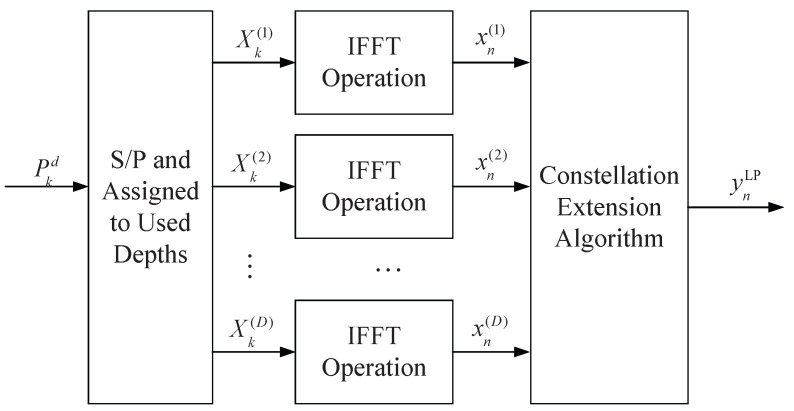
Schematic diagram of constellation extension of PAM.

Consequently, the PAPR reduction algorithm for ASE-DMT based on constellation extension is summarized in Algorithm 1. Meanwhile, the transmitter architecture of the proposed LP-ASE-DMT is illustrated in Figure 2.

In each iteration of the proposed algorithm, only one PAM symbol is selected for constellation extension from the $N\left(1-2^{-D}\right)-1$ candidates, which ensures a low-complexity search process for the optimal solution. Meanwhile, each subsequent iteration builds upon the outcome of the preceding one, thereby permitting each PAM symbol to be extended in multiple levels rather than confining the extension process to a single level. Furthermore, the constellation extension is only performed if the selected PAM symbol leads to a reduced peak amplitude compared to the previous iteration, which ensures the convergence of the proposed algorithm.
**Algorithm 1** PAPR reduction algorithm for ASE-DMT based on constellation extension.Initialize ${\hat{x}}_{n}^{\left(d,0\right)}=x_{n}^{\left(d\right)}$, $b_{0}=\min_{n}\left\{y_{n}\right\}$ and m=0. According to Equations (8) and (11), compute $w_{i_{d},n}^{\left(d\right)}$ and perform iterative operations:**Repeat**Set m=m+1.Compute the constellation-extended signal ${\tilde{x}}_{i_{d},n}^{\left(d,m\right)}$ based on Equation (12).Compute the ASE-DMT signal ${\tilde{y}}_{i_{d},n}^{\left(d,m\right)}$ according to Equation (13).Based on Equations (14) and (15), calculate the peak value $b_{m}$ and determine the optimal extension configurations $d^{*}$ and $i_{d^{*}}^{*}$.Update the time-domain signal ${\hat{x}}_{n}^{\left(d,m\right)}$ according to Equation (16).**Until** $b_{m}>b_{m-1}$.The LP-ASE-DMT signal is given by $y_{n}^{\mathrm{LP}}={\tilde{y}}_{i_{d^{*}}^{*},n}^{\left(d^{*},m-1\right)},\;n=0,1,\ldots ,N-1$.

### 3.2. Complexity Analysis

The complexity analysis of the proposed constellation extension scheme is provided in this section. In the proposed scheme, a total of $N\left(1-2^{-D}\right)-1$ PAM symbols are designated as candidates for constellation extension. The time-domain signal after performing a single-level constellation extension on each PAM symbol is first computed according to Equations (12) and (13) in each iteration, which requires a total of $DN^{2}\left(1-2^{-D}\right)-DN$ additions. Furthermore, comparison operations are performed to determine the minimum peak amplitude, which results in $N^{2}\left(1-2^{-D}\right)-N-1$ comparisons. Therefore, compared to the original ASE-DMT, the proposed LP-ASE-DMT introduces an additional computational complexity of $DN^{2}\left(1-2^{-D}\right)-DN$ additions and $N^{2}\left(1-2^{-D}\right)-N-1$ comparisons at the receiver.

Furthermore, the complexity of the conventional constellation extension scheme based on the theory of compressed sensing (CS) is also provided for comparison [26,27]. In the CS-based scheme, a linear optimization problem involving approximately $N\left(1-2^{-D}\right)-1$ variables is first solved. When the interior-point method is used, approximately $\frac{1}{2}\log_{2}\epsilon \sqrt{N\left(1-2^{-D}\right)}N^{3}{\left(1-2^{-D}\right)}^{3}$ multiplications and additions are required to solve the problem, where ϵ denotes the precision [28]. Furthermore, a set of candidate solutions is randomly generated, and the solution corresponding to the minimum peak amplitude is selected. Consequently, a total of $N_{r}M_{r}DN$ additions and $N_{r}N-1$ comparisons are involved, where $N_{r}$ and $M_{r}$ denotes the number of candidate solutions and PAM symbols performing the constellation extension, respectively. As a result, the complexity of the conventional CS-based scheme is characterized as $\frac{1}{2}\log_{2}\epsilon \sqrt{N\left(1-2^{-D}\right)}N^{3}{\left(1-2^{-D}\right)}^{3}$ multiplications, $\frac{1}{2}\log_{2}\epsilon \sqrt{N\left(1-2^{-D}\right)}N^{3}{\left(1-2^{-D}\right)}^{3}+N_{r}M_{r}DN$ additions, and $N_{r}N-1$ comparisons. Since the value of *N* is significantly larger than that of other parameters, it dominates the overall computational complexity. It is observed that the multiplication and addition operations in the conventional constellation extension scheme are on the order of $O(N^{3}\sqrt{N})$ while the addition and comparison operations in the proposed scheme are on the order of $O(N^{2})$. Meanwhile, since the computational cost of multiplication is substantially higher than that of addition and comparison, the complexity of the proposed scheme is much lower than the conventional CS-based scheme.

## 4. Receiver Design of LP-ASE-DMT

At the transmitter, the optical signal captured by the photodetector is converted into an electrical signal, which is subsequently transformed into the frequency domain via a FFT operation. After frequency-domain equalization, the received signal can be expressed as

(17) $$Y_{k}^{\mathrm{receiver}}=Y_{k}^{\mathrm{LP}}+Z_{k},\;k=0,1,\ldots ,N-1,$$

where $Y_{k}^{\mathrm{LP}}$ denotes the frequency-domain signal of $y_{n}^{\mathrm{LP}}$, and $Z_{k}$ represents the additive white Gaussian noise (AWGN) introduced after frequency-domain equalization.

Furthermore, the PAM symbols of the first depth, which are conveyed at the imaginary components of the subcarriers, are initially detected. The imaginary part of $Y_{k}^{\mathrm{receiver}}$ can be formulated as

(18) ImYkreceiver=ImYkLP+ImZk=X^k(1)+ImZk.

It can be observed that the imaginary part of the received signal, ImYkreceiver, encapsulates the frequency-domain signal ${\hat{X}}_{k}^{\left(1\right)}$ resulting from the constellation extension process. In order to eliminate the influence introduced by the constellation extension, a modulo operation can be applied to ${\hat{X}}_{k}^{\left(1\right)}$. The explicit mathematical formulation is presented as follows:

(19) modImYkreceiver,Δk(1)=jPk(1)+Im(Zk),k=1,2,…,N2−1

**Figure 3:**
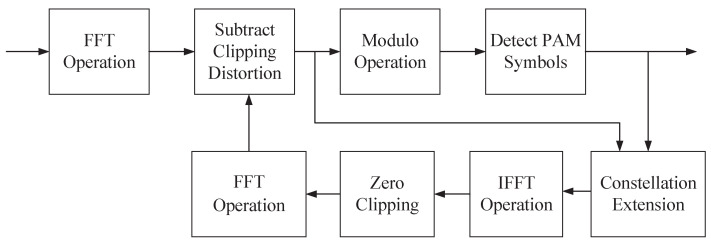
Receiver architecture of the proposed LP-ASE-DMT scheme.

Consequently, PAM symbol detection can be directly performed on the signal after modulo operation, i.e., modImYkreceiver,Δk(1). Upon completing the symbol detection of the first depth, it is imperative to further reconstruct the original clipped signal and eliminate the corresponding clipping noise, so as to facilitate the successive detection of signals in subsequent depths. During this process, the detected PAM symbols are utilized to reconstruct the clipped signal. Concurrently, the imaginary part of the received signal, ImYkreceiver, is exploited to determine whether constellation extension has occurred, which provides the basis for subsequent recovery of the clipping noise. Once the clipping noise is accurately reconstructed, it is subtracted from the received signal $Y_{k}^{\mathrm{receiver}}$ to mitigate its interference. Subsequently, the real part of the updated signal is employed to extract the PAM symbols of the next depth through an analogous detection procedure. Specifically, as depicted in Figure 3, the receiver architecture of the proposed LP-ASE-DMT scheme incorporates only a low-complexity modulo and constellation extension module. Compared to the conventional ASE-DMT receiver, the proposed architecture enables efficient detection without significantly increasing the overall receiver complexity.

## 5. Simulation Results and Discussion

In this section, a comprehensive analysis and discussion of the simulation results is provided. The PAPR characteristics of the proposed scheme are first examined. Subsequently, its BER performance and power efficiency are thoroughly evaluated under nonlinear transmission conditions. In the simulation setup, the number of subcarriers in ASE-DMT is configured to 128. A nonlinear emission model with dual-sided clipping is employed to evaluate the transmitter nonlinearity. To quantitatively assess the degree of nonlinearity, the clipping ratio is defined as $\tau =20\log_{10}\left(\frac{A}{\sigma }\right)$, where *A* denotes the upper limit imposed by the nonlinear emitter, and σ represents the standard deviation of the ASE-DMT signal.

**Figure 4:**
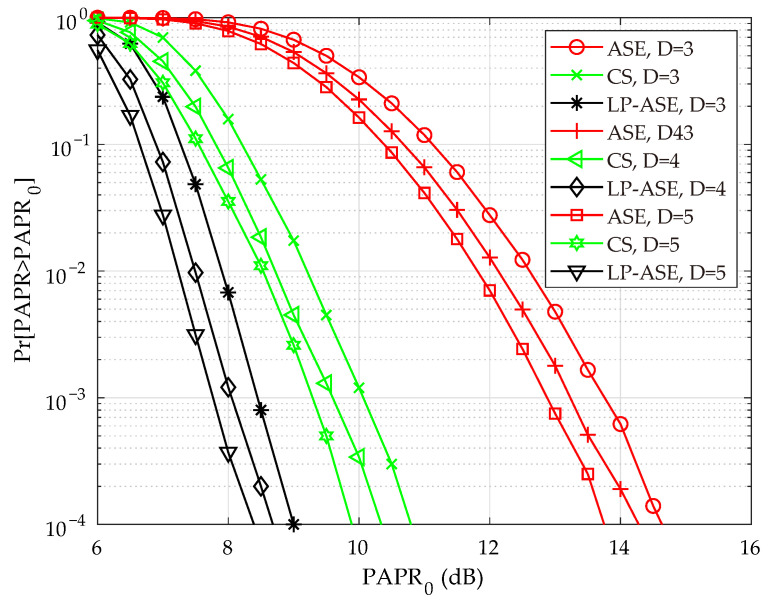
CCDF curves of the proposed LP-ASE-DMT, ASE-DMT, and the CS-based constellation extension scheme.

Figure 4 presents the complementary cumulative distribution function (CCDF) curves of PAPR for the proposed LP-ASE-DMT under different number of depths. For comparison, the PAPR performance of the conventional ASE-DMT and CS-based constellation extension schemes are also provided. In the simulation, 4-PAM is employed for bit-to-symbol mapping. The number of the candidate solutions randomly generated is set to 40 in the CS-based scheme. It is observed from Figure 4 that, for a given PAPR threshold, the probability that the PAPR of the proposed LP-ASE-DMT exceeds the threshold is significantly lower than that of the original ASE-DMT and CS-based schemes. It clearly indicates that the proposed LP-ASE-DMT achieves substantial PAPR reduction compared to its counterparts. Specifically, when the the target CCDF probability is set to $10^{-4}$, a PAPR reduction of approximately 5.5 dB is observed for LP-ASE-DMT, thereby substantially improving the system’s resilience to nonlinear distortion.

**Figure 5:**
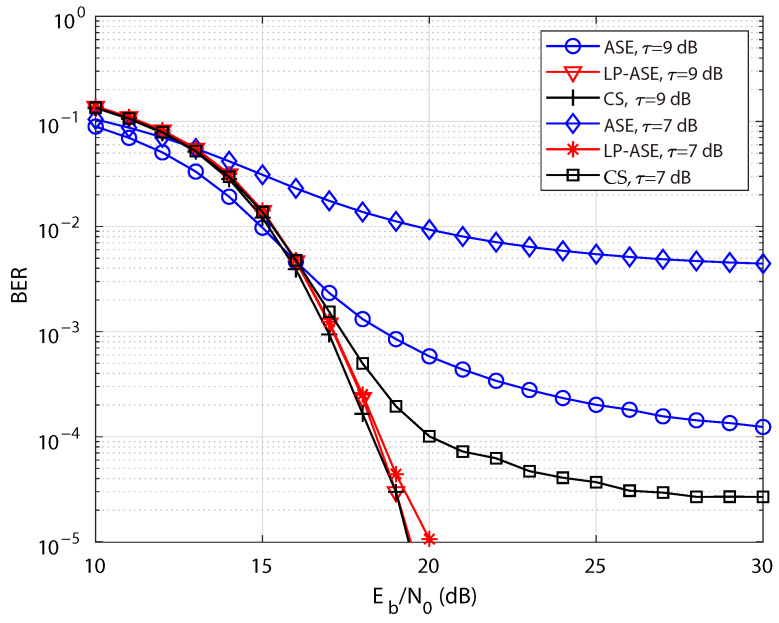
BER performance of LP-ASE-DMT, ASE-MDT, and the CS-based scheme under different nonlinearity conditions.

Figure 5 illustrates the BER performance of LP-ASE-DMT, ASE-DMT and the CS-based scheme under different nonlinearity conditions, where 4-PAM is adopted for symbol mapping, and the number of depths is set to D=4. A relatively small value of τ implies a diminishing disparity between the upper clipping threshold *A* and the standard deviation of the ASE-DMT signal σ, thereby exacerbating the system’s nonlinear distortion. As shown in Figure 5, due to its inherently high PAPR and severe error propagation introduced by the SIC-based receiver, the original ASE-DMT scheme suffers from notable BER performance degradation under nonlinearity conditions. With the aggravation of system nonlinearity, the BER performance of ASE-DMT exhibits a marked degradation. Meanwhile, as $E_{b}/N_{0}$ increases, BER is primarily determined by the system’s nonlinear distortion rather than random noise. Since such nonlinear distortion is independent of $E_{b}/N_{0}$, further increasing $E_{b}/N_{0}$ does not lead to any improvement in BER, resulting in a distinct BER floor. Furthermore, compared to the original ASE-DMT, the proposed LP-ASE-DMT and the CS-based scheme achieves a substantial reduction in PAPR, thus yielding a pronounced improvement in BER performance. Moreover, owing to its significantly lower PAPR compared to the CS-based scheme, the proposed LP-ASE-DMT achieves superior BER performance under the severe nonlinearity condition of τ=7 dB. Specifically, under a bit energy-to-noise power ratio of $E_{b}/N_{0}=18\;\mathrm{dB}$ and a clipping threshold of τ=7dB, the BERs of ASE-DMT, LP-ASE-DMT and the CS-based scheme are $1.37\times 10^{-2}$, $2.52\times 10^{-4}$ and $4.97\times 10^{-4}$, respectively. Therefore, LP-ASE-DMT achieves a BER improvement of over two orders of magnitude compared to the conventional ASE-DMT scheme.

**Figure 6:**
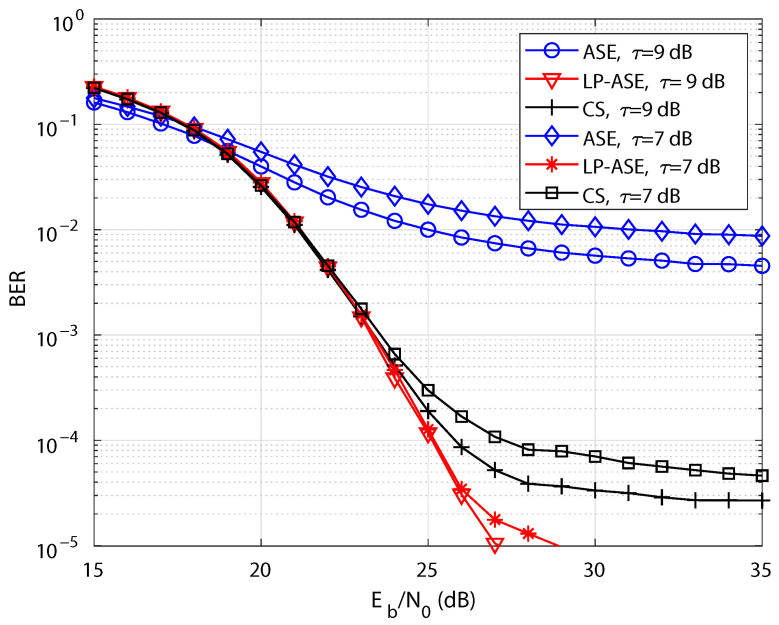
BER performance of LP-ASE-DMT, ASE-DMT, and the CS-based scheme under different nonlinearity conditions and diffused optical wireless channels.

Furthermore, the diffused optical wireless channel incorporating the effects of multi-path propagation is considered to comprehensively evaluate the BER performance [29,30]. The ceiling bounce model is employed to characterize the diffused channel. In alignment with [29], the number of taps is set to 64 in the simulation. Meanwhile, delay spread is randomly chosen between 0.2 and 7 ns, and the sampling period is set to 2 ns. Figure 6 illustrates the BER curves of LP-ASE-DMT, ASE-DMT, and the CS-based scheme under different nonlinearity conditions and diffused optical wireless channels. Benefiting from its superior PAPR performance, the proposed LP-ASE-DMT demonstrates markedly improved BER performance compared to ASE-DMT and the CS-based scheme under diffused optical wireless channels.

**Figure 7:**
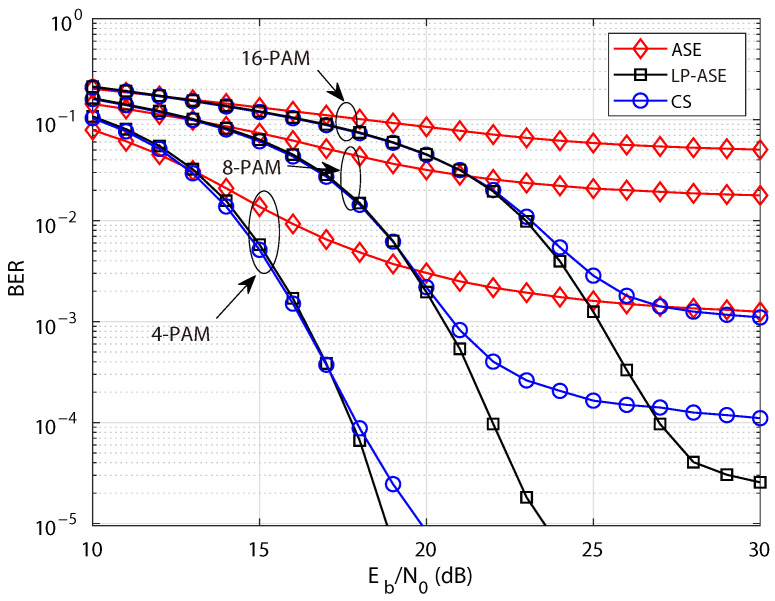
BER performance of LP-ASE-DMT, ASE-DMT, and the CS-based scheme with various modulation orders under the nonlinearity condition of τ=8 dB.

The BER performance of LP-ASE-DMT, ASE-DMT and the CS-based scheme under the nonlinearity condition are investigated for various modulation orders in Figure 7, where the clipping ratio and the number of depths are set to τ=8dB and D=3, respectively. It can be observed that as the modulation order increases, the BER performance progressively degrades, indicating that higher-order PAM is more susceptible to nonlinear distortion. Meanwhile, the proposed LP-ASE-DMT scheme exhibits consistently superior BER performance compared to ASE-DMT and the CS-based scheme across different modulation orders. Moreover, for the target bit error rate of ${\mathrm{BER}}_{\mathrm{target}}=2\times 10^{-3}$, the conventional ASE-DMT scheme employing 4-PAM is capable of satisfying the performance requirement. However, as the modulation order increases, ASE-DMT becomes incapable of achieving the desired BER due to the pronounced impact of nonlinear distortions. In contrast, the proposed LP-ASE-DMT scheme, by leveraging higher-order modulation in conjunction with enhanced resilience to nonlinearity, can successfully meet the target BER criterion. Furthermore, owing to the superior spectral efficiency facilitated by higher-order PAM, the proposed LP-ASE-DMT scheme exhibits a markedly enhanced capability to support high data rates, thereby outperforming the conventional ASE-DMT in terms of spectral efficiency.

**Figure 8:**
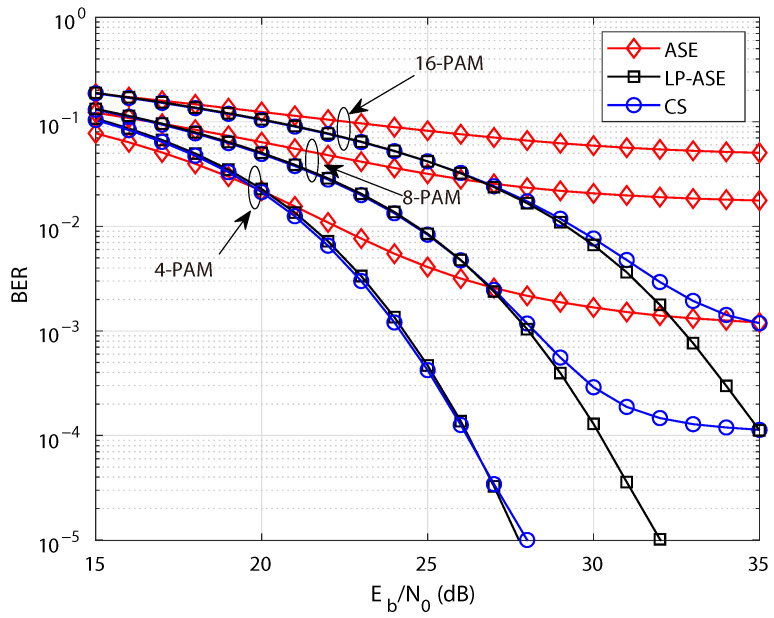
BER performance of LP-ASE-DMT, ASE-DMT, and the CS-based scheme with various modulation orders under diffused optical wireless channels.

Furthermore, the BER performance of LP-ASE-DMT, ASE-DMT and the CS-based scheme with various modulation orders is investigated under diffused optical wireless channels, as illustrated in Figure 8. The clipping ratio is set to τ=8 dB, and the modulation depth is fixed at D=3 in the simulation. Similarly, it is observed that under diffused optical wireless channels, the schemes employing high-order PAM exhibit more pronounced BER degradation due to increased sensitivity to nonlinear distortion. Moreover, benefiting from its significantly lower PAPR, the proposed LP-ASE-DMT achieves much better BER performance than ASE-DMT and the CS-based scheme, particularly in the case of high-order PAM symbols.

**Figure 9:**
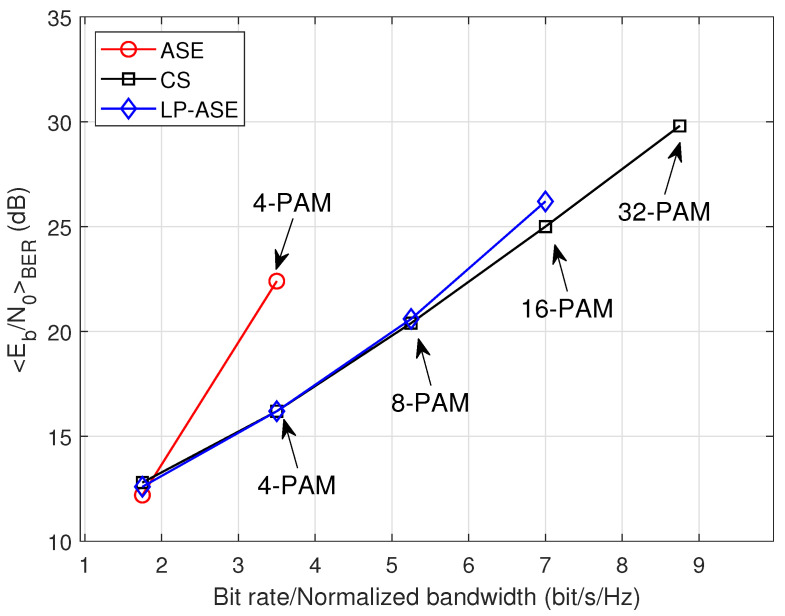
Required $E_{b}/N_{0}$ to achieve the target BER of $2\times 10^{-3}$ for LP-ASE-DMT and ASE-DMT under the nonlinearity conditions.

Figure 9 presents the required $E_{b}/N_{0}$ to achieve the target BER of $2\times 10^{-3}$ for LP-ASE-DMT, ASE-DMT, and the CS-based scheme under the nonlinearity condition. In the simulation, the clipping ratio is configured as τ=8dB, and the number of depths is set to D=3. Owing to the pronounced nonlinear distortion resulting from the high PAPR, the conventional ASE-DMT necessitates a significantly higher $E_{b}/N_{0}$ to attain the target BER compared to the proposed LP-ASE-DMT and the CS-based scheme. Moreover, as the modulation order increases, the CS-based scheme requires progressively higher $E_{b}/N_{0}$ compared to LP-ASE-DMT, which highlights the superior power efficiency achieved by the proposed architecture. More importantly, the conventional ASE-DMT and CS-based schemes support a maximum bit rate/normalized bandwidth of 3.5 bit/s/Hz and 7 bit/s/Hz, respectively. In contrast, the proposed LP-ASE-DMT scheme is capable of achieving up to 8.75 bit/s/Hz, thereby demonstrating a marked improvement in spectral efficiency.

## 6. Conclusions

To address the inherent limitation of high PAPR in ASE-DMT, a novel LP-ASE-DMT is proposed with the aid of the constellation extension in the paper. To be specific, given the intricate signal superposition structure resulting from multi-depth modulation in ASE-DMT, a progressive constellation extension algorithm is innovatively developed to achieve the substantial PAPR reduction. Compared to the conventional CS-based scheme, the proposed scheme enables multi-level extension to fully exploit the degree of freedom, while significantly reducing the computational complexity. Furthermore, a dedicated receiver architecture is designed for the proposed LP-ASE-DMT, in which a low-complexity modulo operation is adopted to eliminate the impact of constellation extension. As a result, the receiver complexity remains comparable to that of the conventional ASE-DMT scheme, without incurring significant additional computational overhead. Simulation results demonstrate that the proposed LP-ASE-DMT achieves a substantial PAPR reduction compared to its counterparts. In particular, at a CCDF probability of $10^{-4}$, a PAPR reduction of approximately 5.5 dB is observed for LP-ASE-DMT. Owing to its superior PAPR performance, LP-ASE-DMT exhibits notable performance advantages under nonlinearity conditions with respect to multiple metrics, including BER, spectral efficiency, and power efficiency.

## Data Availability

Data available upon request.
